# Supplementary material for: Fertility plans in the early times of the COVID-19 pandemic: The role of occupational and financial uncertainty in Italy
Source: PLoS One. 2022 Dec 8;17(12):e0271384. doi: 10.1371/journal.pone.0271384 (PMC9731473; doi:10.1371/journal.pone.0271384)
Supplement: S1 Appendix — S1_1 Table. Ordered logit model for the fertility intentions at 12 months, by gender (Regular waves, 2016 and 2020). S1_2 Table. Multinomial models for the intention of still-planning, postponing or abandoning the pre-pandemic fertility plan, for women (COVID waves, March and October 2020). S1_3 Table. Multinomial models for the intention of still-planning, postponing or abandoning the pre-pandemic fertility plan, for men (COVID waves, March and October 2020). S1_4 Table. Multinomial models for the intention of still-planning, postponing or abandoning the pre-pandemic fertility plan with survey wave as mediator, for women (COVID waves, March and October 2020). S1_5 Table. Multinomial models for the intention of still-planning, postponing or abandoning the pre-pandemic fertility plan with survey wave as mediator, for men (COVID waves, March and October 2020). S1_1 Text. The selection into the “no plans” group. S1_6 Table. Logit models for the probability of not planning to have a child in the 2020, by including variables on income and occupation vulnerability due to COVID-19, in 2020 (Data source: Rapporto Giovani. March–October 2020). Robustness checks: S1_2 Text. Stronger and weaker retrospective fertility intentions. S1_7 Table. Multinomial models for the intention of still-planning, postponing or abandoning the pre-pandemic fertility plan, in the restricted and the enlarged samples (Data source: Rapporto Giovani COVID survey, March 2020 and October 2020). S1_3 Text. Clustering occupational categories. S1_1 Fig. Predicted probabilities of changes or confirmations of pre-COVID fertility plans in March and October 2020 across occupational conditions (confidence intervals for pair-wise comparisons at the 10% significance level). Including full-time students in the analysed samples. S1_2 Fig. Predicted probabilities of intending to have a(nother) child (answer: “surely yes”) in the next 12 months by occupational condition in 2016 and 2020 and by gender, including full [file pone.0271384.s001.docx]

**Appendix**

**Table S1_1. Ordered logit model for the fertility intentions at 12 months, by gender (Regular waves, 2016 and 2020)**

|  | **Women** | | **Men** | |
| --- | --- | --- | --- | --- |
|  | **Coef.** | **p-value** | **Coef.** | **p-value** |
| **Surely not** |  |  |  |  |
| Age class (ref. 18-24) |  |  |  |  |
| 18-24 | -0.710 | 0.016 | -0.111 | 0.817 |
| 30-34 | 0.708 | 0.000 | 0.691 | 0.011 |
|  |  |  |  |  |
| Tertiary education | -0.435 | 0.001 | -0.022 | 0.913 |
|  |  |  |  |  |
| Occupational status (ref. Employees) |  |  |  |  |
| NEET | -0.007 | 0.977 | -0.053 | 0.920 |
| self-employed & temporary | 0.622 | 0.059 | 0.741 | 0.221 |
|  |  |  |  |  |
| Wave (ref. 2016) |  |  |  |  |
| 2020 | 0.183 | 0.363 | 0.401 | 0.155 |
|  |  |  |  |  |
| Occupational status#wave |  |  |  |  |
| NEET#2020 | -0.290 | 0.465 | 0.241 | 0.759 |
| self-employed & temporary#2020 | -0.399 | 0.394 | -0.142 | 0.855 |
|  |  |  |  |  |
| Married/cohabiting | 0.982 | 0.000 | 1.417 | 0.005 |
| With children | -0.139 | 0.519 | -0.101 | 0.842 |
| Constant | 0.241 | 0.414 | 0.148 | 0.761 |
|  |  |  |  |  |
| **Probably not** |  |  |  |  |
| Age class (ref. 18-24) |  |  |  |  |
| 18-24 | -0.224 | 0.462 | -0.661 | 0.174 |
| 30-34 | 0.526 | 0.000 | 0.477 | 0.056 |
|  |  |  |  |  |
| Tertiary education | -0.384 | 0.001 | -0.100 | 0.540 |
|  |  |  |  |  |
| Occupational status (ref. Employees) |  |  |  |  |
| NEET | 0.040 | 0.842 | 0.030 | 0.957 |
| self-employed & temporary | 0.218 | 0.594 | 1.000 | 0.093 |
|  |  |  |  |  |
| Wave (ref. 2016) |  |  |  |  |
| 2020 | 0.162 | 0.306 | 0.204 | 0.356 |
|  |  |  |  |  |
| Occupational status#wave |  |  |  |  |
| NEET#2020 | -0.462 | 0.159 | 0.423 | 0.566 |
| self-employed & temporary#2020 | -0.651 | 0.227 | -0.468 | 0.527 |
|  |  |  |  |  |
| Married/cohabiting | 0.878 | 0.000 | 1.153 | 0.000 |
| With children | 0.117 | 0.484 | 0.198 | 0.460 |
| Constant | -1.160 | 0.000 | -2.128 | 0.000 |
|  |  |  |  |  |
| **Probably yes** |  |  |  |  |
| Age class (ref. 18-24) |  |  |  |  |
| 18-24 | -0.059 | 0.908 | -1.148 | 0.199 |
| 30-34 | 0.322 | 0.164 | 0.711 | 0.101 |
|  |  |  |  |  |
| Tertiary education | -0.443 | 0.009 | 0.230 | 0.471 |
|  |  |  |  |  |
| Occupational status (ref. Employees) |  |  |  |  |
| NEET | 0.143 | 0.636 | -0.371 | 0.746 |
| self-employed & temporary | 0.510 | 0.286 | 1.663 | 0.024 |
|  |  |  |  |  |
| Wave (ref. 2016) |  |  |  |  |
| 2020 | 0.501 | 0.026 | 0.220 | 0.573 |
|  |  |  |  |  |
| Occupational status#wave |  |  |  |  |
| NEET#2020 | -0.945 | 0.067 | 0.758 | 0.589 |
| self-employed & temporary#2020 | -2.424 | 0.001 | -1.761 | 0.065 |
|  |  |  |  |  |
| Married/cohabiting | 0.928 | 0.000 | 1.573 | 0.000 |
| With children | -0.194 | 0.370 | -0.396 | 0.326 |
| Constant | -2.686 | 0.000 | -5.658 | 0.000 |

**Table S1_2. Multinomial models for the intention of still-planning, postponing or abandoning the pre-pandemic fertility plan, for women (COVID waves, March and October 2020)**

|  | **Model 1** | | **Model 2** | | **Model 3** | | **Model 4** | |
| --- | --- | --- | --- | --- | --- | --- | --- | --- |
|  | **Coeff** | **p-value** | **Coeff** | **p-value** | **Coeff** | **p-value** | **Coeff** | **p-value** |
| **still-planning (ref)** |  |  |  |  |  |  |  |  |
| **postponing** |  |  |  |  |  |  |  |  |
| Age class |  |  |  |  |  |  |  |  |
| 18-24 | -0.075 | 0.862 | -0.075 | 0.862 | -0.055 | 0.898 | -0.046 | 0.916 |
| 30-34 | -0.397 | 0.174 | -0.402 | 0.169 | -0.418 | 0.153 | -0.437 | 0.137 |
|  |  |  |  |  |  |  |  |  |
| Tertiary education | 0.203 | 0.464 | 0.194 | 0.486 | 0.235 | 0.392 | 0.242 | 0.377 |
|  |  |  |  |  |  |  |  |  |
| Married/cohabiting | -0.410 | 0.152 | -0.426 | 0.138 | -0.425 | 0.136 | -0.434 | 0.130 |
| With children | -0.379 | 0.174 | -0.379 | 0.174 | -0.366 | 0.188 | -0.366 | 0.190 |
| October 2020 (ref. March 2020) | -0.394 | 0.136 | -0.403 | 0.127 | -0.348 | 0.193 | -0.340 | 0.204 |
| High spread SARS-CoV-2 region | -0.109 | 0.677 | -0.101 | 0.701 | -0.106 | 0.686 | -0.102 | 0.697 |
|  |  |  |  |  |  |  |  |  |
| Occupational status (ref. Employees) |  |  |  |  |  |  |  |  |
| NEET | -0.012 | 0.971 | 0.008 | 0.980 | 0.005 | 0.988 | 0.009 | 0.977 |
| Self-employed & temporary | 0.083 | 0.840 | 0.035 | 0.932 | 0.112 | 0.783 | 0.103 | 0.838 |
|  |  |  |  |  |  |  |  |  |
| Occupation at risk |  |  | 0.234 | 0.362 |  |  |  |  |
| Financial condition already worsen |  |  |  |  | 0.192 | 0.461 |  |  |
| Income at risk |  |  |  |  |  |  | 0.258 | 0.328 |
|  |  |  |  |  |  |  |  |  |
| Constant | 1.082 | 0.039 | 0.975 | 0.069 | 0.871 | 0.099 | 0.918 | 0.095 |
| **abandoning** |  |  |  |  |  |  |  |  |
| Age class |  |  |  |  |  |  |  |  |
| 18-24 | 0.036 | 0.935 | 0.028 | 0.950 | 0.124 | 0.781 | 0.168 | 0.709 |
| 30-34 | -0.565 | 0.071 | -0.576 | 0.067 | -0.702 | 0.027 | -0.668 | 0.036 |
|  |  |  |  |  |  |  |  |  |
| Tertiary education | -0.645 | 0.045 | -0.645 | 0.045 | -0.477 | 0.075 | -0.577 | 0.075 |
|  |  |  |  |  |  |  |  |  |
| Married/cohabiting | -0.632 | 0.041 | -0.677 | 0.030 | -0.739 | 0.019 | -0.702 | 0.026 |
| With children | -0.337 | 0.276 | -0.325 | 0.296 | -0.264 | 0.399 | -0.321 | 0.306 |
| October 2020 (ref. March 2020) | -0.975 | 0.001 | -1.024 | 0.001 | -0.752 | 0.012 | -0.823 | 0.006 |
| High spread SARS-CoV-2 region | -0.604 | 0.038 | -0.599 | 0.041 | -0.658 | 0.027 | -0.626 | 0.034 |
|  |  |  |  |  |  |  |  |  |
| Occupational status (ref. Employees) |  |  |  |  |  |  |  |  |
| NEET | 0.326 | 0.325 | 0.376 | 0.260 | 0.470 | 0.150 | 0.317 | 0.343 |
| Self-employed & temporary | 0.337 | 0.439 | 0.237 | 0.590 | 0.458 | 0.295 | 0.282 | 0.525 |
|  |  |  |  |  |  |  |  |  |
| Occupation at risk |  |  | 0.581 | 0.042 |  |  |  |  |
| Financial condition already worsen |  |  |  |  | 0.962 | 0.001 |  |  |
| Income at risk |  |  |  |  |  |  | 0.850 | 0.004 |
|  |  |  |  |  |  |  |  |  |
| Constant | 2.255 | 0.000 | 1.998 | 0.000 | 1.152 | 0.041 | 1.669 | 0.005 |

**Table S1_3. Multinomial models for the intention of still-planning, postponing or abandoning the pre-pandemic fertility plan, for men (COVID waves, March and October 2020)**

|  | **Model 1** | | **Model 2** | | **Model 3** | | **Model 4** | |
| --- | --- | --- | --- | --- | --- | --- | --- | --- |
|  | **Coeff** | **p-value** | **Coeff** | **p-value** | **Coeff** | **p-value** | **Coeff** | **p-value** |
| **still-planning (ref)** |  |  |  |  |  |  |  |  |
| **postponing** |  |  |  |  |  |  |  |  |
| Age class |  |  |  |  |  |  |  |  |
| 18-24 | 0.176 | 0.639 | 0.178 | 0.635 | 0.178 | 0.635 | 0.204 | 0.587 |
| 30-34 | -0.281 | 0.374 | -0.275 | 0.385 | -0.255 | 0.418 | -0.282 | 0.372 |
|  |  |  |  |  |  |  |  |  |
| Tertiary education | -0.037 | 0.883 | -0.038 | 0.879 | -0.042 | 0.868 | -0.023 | 0.928 |
|  |  |  |  |  |  |  |  |  |
| Married/cohabiting | -0.742 | 0.009 | -0.742 | 0.009 | -0.744 | 0.009 | -0.703 | 0.015 |
| With children | -0.358 | 0.241 | -0.357 | 0.243 | -0.342 | 0.259 | -0.392 | 0.203 |
| October 2020 (ref. March 2020) | -0.491 | 0.059 | -0.481 | 0.069 | -0.485 | 0.063 | -0.436 | 0.101 |
| High spread SARS-CoV-2 region | 0.136 | 0.600 | 0.137 | 0.599 | 0.135 | 0.603 | 0.141 | 0.588 |
|  |  |  |  |  |  |  |  |  |
| Occupational status (ref. Employees) |  |  |  |  |  |  |  |  |
| NEET | -0.014 | 0.973 | -0.003 | 0.994 | -0.046 | 0.913 | -0.022 | 0.959 |
| Self-employed & temporary | 0.559 | 0.141 | 0.561 | 0.140 | 0.579 | 0.127 | 0.553 | 0.147 |
|  |  |  |  |  |  |  |  |  |
| Occupation at risk |  |  | -0.049 | 0.849 |  |  |  |  |
| Financial condition already worsen |  |  |  |  | 0.011 | 0.968 |  |  |
| Income at risk |  |  |  |  |  |  | 0.268 | 0.336 |
|  |  |  |  |  |  |  |  |  |
| Constant | 1.389 | 0.003 | 1.399 | 0.003 | 1.416 | 0.003 | 1.213 | 0.016 |
| **abandoning** |  |  |  |  |  |  |  |  |
| Age class |  |  |  |  |  |  |  |  |
| 18-24 | -0.287 | 0.523 | -0.291 | 0.518 | -0.312 | 0.488 | -0.245 | 0.588 |
| 30-34 | -0.198 | 0.590 | -0.218 | 0.554 | -0.259 | 0.481 | -0.231 | 0.530 |
|  |  |  |  |  |  |  |  |  |
| Tertiary education | -0.265 | 0.377 | -0.251 | 0.405 | -0.208 | 0.493 | -0.232 | 0.443 |
|  |  |  |  |  |  |  |  |  |
| Married/cohabiting | -0.340 | 0.302 | -0.344 | 0.297 | -0.349 | 0.289 | -0.237 | 0.480 |
| With children | -0.551 | 0.131 | -0.543 | 0.135 | -0.490 | 0.178 | -0.646 | 0.081 |
| October 2020 (ref. March 2020) | -1.071 | 0.001 | -1.117 | 0.001 | -1.013 | 0.002 | -0.929 | 0.005 |
| High spread SARS-CoV-2 region | 0.185 | 0.545 | 0.184 | 0.547 | 0.203 | 0.507 | 0.201 | 0.513 |
|  |  |  |  |  |  |  |  |  |
| Occupational status (ref. Employees) |  |  |  |  |  |  |  |  |
| NEET | 0.149 | 0.760 |  |  | 0.067 | 0.890 | 0.102 | 0.836 |
| Self-employed & temporary | 1.303 | 0.001 |  |  | 1.342 | 0.001 | 1.284 | 0.002 |
|  |  |  |  |  |  |  |  |  |
| Occupation at risk |  |  | 0.233 | 0.443 |  |  |  |  |
| Financial condition already worsen |  |  |  |  | 0.540 | 0.082 |  |  |
| Income at risk |  |  |  |  |  |  | 0.655 | 0.039 |
|  |  |  |  |  |  |  |  |  |
| Constant | 1.411 | 0.010 | 1.345 | 0.015 | 1.214 | 0.030 | 0.966 | 0.101 |

**Table S1_4. Multinomial models for the intention of still-planning, postponing or abandoning the pre-pandemic fertility plan with survey wave as mediator, for women (COVID waves, March and October 2020)**

|  | **Model 1** | | **Model 2** | | **Model 3** | | **Model 4** | |
| --- | --- | --- | --- | --- | --- | --- | --- | --- |
|  | **Coeff.** | **p-value** | **Coeff.** | **p-value** | **Coeff.** | **p-value** | **Coeff.** | **p-value** |
| **Still-planning** |  |  |  |  |  |  |  |  |
| **Postponing** |  |  |  |  |  |  |  |  |
| Age class |  |  |  |  |  |  |  |  |
| 18-24 | -0.045 | 0.917 | -0.084 | 0.847 | -0.019 | 0.965 | -0.071 | 0.870 |
| 30-34 | -0.393 | 0.179 | -0.419 | 0.154 | -0.435 | 0.140 | -0.394 | 0.178 |
|  |  |  |  |  |  |  |  |  |
| Tertiary education | 0.186 | 0.505 | 0.216 | 0.440 | 0.220 | 0.432 | 0.189 | 0.496 |
|  |  |  |  |  |  |  |  |  |
| Married/cohabiting | -0.412 | 0.153 | -0.404 | 0.159 | -0.430 | 0.135 | -0.418 | 0.146 |
| With children | -0.386 | 0.169 | -0.371 | 0.183 | -0.368 | 0.189 | -0.379 | 0.173 |
| High spread COVID-19 region | -0.116 | 0.661 | -0.089 | 0.735 | -0.098 | 0.709 | -0.090 | 0.733 |
|  |  |  |  |  |  |  |  |  |
| Occupational status |  |  |  |  |  |  |  |  |
| NEET | 0.294 | 0.491 | -0.035 | 0.916 | -0.033 | 0.922 | 0.001 | 0.997 |
| Self-employed & temporary | 0.682 | 0.303 | 0.093 | 0.820 | 0.076 | 0.853 | 0.034 | 0.934 |
| October 2020 (ref. March 2020) | -0.155 | 0.619 |  |  |  |  |  |  |
| NEET*wave | -0.667 | 0.306 |  |  |  |  |  |  |
| Self-employed & temporary*wave | -0.974 | 0.252 |  |  |  |  |  |  |
|  |  |  |  |  |  |  |  |  |
| Financial condition already worsen |  |  | -0.107 | 0.767 |  |  |  |  |
| October 2020 (ref. March 2020) |  |  | -0.630 | 0.079 |  |  |  |  |
| Worse financial condition*wave |  |  | 0.602 | 0.244 |  |  |  |  |
|  |  |  |  |  |  |  |  |  |
| Income at risk |  |  |  |  | 0.005 | 0.988 |  |  |
| October 2020 (ref. March 2020) |  |  |  |  | -0.562 | 0.111 |  |  |
| Income at risk*wave |  |  |  |  | 0.493 | 0.346 |  |  |
|  |  |  |  |  |  |  |  |  |
| Occupation at risk |  |  |  |  |  |  | 0.127 | 0.723 |
| October 2020 (ref. March 2020) |  |  |  |  |  |  | -0.563 | 0.161 |
| Occupation at risk*wave |  |  |  |  |  |  | 0.258 | 0.619 |
|  |  |  |  |  |  |  |  |  |
| Constant | 0.567 | 0.137 | 0.859 | 0.055 | 1.124 | 0.010 | 0.620 | 0.130 |
|  |  |  |  |  |  |  |  |  |
| **Abandoning** |  |  |  |  |  |  |  |  |
| Age class |  |  |  |  |  |  |  |  |
| 18-24 | 0.075 | 0.868 | 0.169 | 0.708 | 0.213 | 0.641 | 0.007 | 0.987 |
| 30-34 | -0.574 | 0.069 | -0.661 | 0.039 | -0.685 | 0.033 | -0.584 | 0.064 |
|  |  |  |  |  |  |  |  |  |
| Tertiary education | -0.677 | 0.037 | -0.581 | 0.076 | -0.587 | 0.073 | -0.632 | 0.050 |
|  |  |  |  |  |  |  |  |  |
| Married/cohabiting | -0.630 | 0.046 | -0.711 | 0.025 | -0.715 | 0.024 | -0.697 | 0.027 |
| With children | -0.355 | 0.259 | -0.267 | 0.400 | -0.300 | 0.341 | -0.313 | 0.315 |
| High spread COVID-19 region | -0.590 | 0.044 | -0.647 | 0.029 | -0.609 | 0.039 | -0.623 | 0.034 |
|  |  |  |  |  |  |  |  |  |
| Occupational status |  |  |  |  |  |  |  |  |
| NEET | 0.797 | 0.051 | 0.302 | 0.367 | 0.301 | 0.369 | 0.386 | 0.250 |
| Self-employed & temporary | 1.168 | 0.071 | 0.297 | 0.507 | 0.272 | 0.543 | 0.230 | 0.602 |
| October 2020 (ref. March 2020) | -0.446 | 0.216 |  |  |  |  |  |  |
| NEET*wave | -1.385 | 0.055 |  |  |  |  |  |  |
| Self-employed & temporary*wave | -1.789 | 0.064 |  |  |  |  |  |  |
|  |  |  |  |  |  |  |  |  |
| Financial condition already worsen |  |  | 0.765 | 0.044 |  |  |  |  |
| October 2020 (ref. March 2020) |  |  | -1.078 | 0.015 |  |  |  |  |
| Worse financial condition*wave |  |  | 0.500 | 0.397 |  |  |  |  |
|  |  |  |  |  |  |  |  |  |
| Income at risk |  |  |  |  | 0.394 | 0.280 |  |  |
| October 2020 (ref. March 2020) |  |  |  |  | -1.433 | 0.001 |  |  |
| Income at risk*wave |  |  |  |  | 1.210 | 0.040 |  |  |
|  |  |  |  |  |  |  |  |  |
| Occupation at risk |  |  |  |  |  |  | 0.759 | 0.037 |
| October 2020 (ref. March 2020) |  |  |  |  |  |  | -0.630 | 0.172 |
| Occupation at risk*wave |  |  |  |  |  |  | -0.608 | 0.302 |
|  |  |  |  |  |  |  |  |  |
| Constant | 1.058 | 0.007 | 0.859 | 0.055 | 1.124 | 0.010 | 0.884 | 0.039 |

**Table S1_5. Multinomial models for the intention of still-planning, postponing or abandoning the pre-pandemic fertility plan with survey wave as mediator, for men (COVID waves, March and October 2020)**

|  | **Model 1** | | **Model 2** | | **Model 3** | | **Model 4** | |
| --- | --- | --- | --- | --- | --- | --- | --- | --- |
|  | **Coeff.** | **p-value** | **Coeff.** | **p-value** | **Coeff.** | **p-value** | **Coeff.** | **p-value** |
| **Still-planning** |  |  |  |  |  |  |  |  |
| **Postponing** |  |  |  |  |  |  |  |  |
| Age class |  |  |  |  |  |  |  |  |
| 18-24 | 0.203 | 0.590 | 0.178 | 0.634 | 0.207 | 0.582 | 0.137 | 0.716 |
| 30-34 | -0.258 | 0.414 | -0.298 | 0.350 | -0.288 | 0.363 | -0.306 | 0.337 |
|  |  |  |  |  |  |  |  |  |
| Tertiary education | -0.051 | 0.841 | -0.026 | 0.918 | -0.015 | 0.953 | -0.016 | 0.950 |
|  |  |  |  |  |  |  |  |  |
| Married/cohabiting | -0.765 | 0.008 | -0.743 | 0.010 | -0.707 | 0.015 | -0.758 | 0.008 |
| With children | -0.371 | 0.226 | -0.356 | 0.246 | -0.389 | 0.206 | -0.319 | 0.300 |
| High spread COVID-19 region | 0.134 | 0.608 | 0.146 | 0.577 | 0.145 | 0.578 | 0.158 | 0.545 |
|  |  |  |  |  |  |  |  |  |
| Occupational status |  |  |  |  |  |  |  |  |
| NEET | 0.271 | 0.616 | -0.039 | 0.926 | -0.029 | 0.946 | 0.030 | 0.944 |
| Self-employed & temporary | 1.218 | 0.068 | 0.564 | 0.139 | 0.555 | 0.145 | 0.554 | 0.146 |
| October 2020 (ref. March 2020) | -0.329 | 0.268 |  |  |  |  |  |  |
| NEET*wave | -0.724 | 0.395 |  |  |  |  |  |  |
| Self-employed & temporary*wave | -1.009 | 0.223 |  |  |  |  |  |  |
|  |  |  |  |  |  |  |  |  |
| Financial condition already worsen |  |  | 0.273 | 0.448 |  |  |  |  |
| October 2020 (ref. March 2020) |  |  | -0.311 | 0.308 |  |  |  |  |
| Worse financial condition*wave |  |  | -0.629 | 0.264 |  |  |  |  |
|  |  |  |  |  |  |  |  |  |
| Income at risk |  |  |  |  | 0.330 | 0.347 |  |  |
| October 2020 (ref. March 2020) |  |  |  |  | -0.389 | 0.203 |  |  |
| Income at risk*wave |  |  |  |  | -0.176 | 0.762 |  |  |
|  |  |  |  |  |  |  |  |  |
| Occupation at risk |  |  |  |  |  |  | -0.313 | 0.346 |
| October 2020 (ref. March 2020) |  |  |  |  |  |  | -0.894 | 0.034 |
| Occupation at risk*wave |  |  |  |  |  |  | 0.666 | 0.207 |
|  |  |  |  |  |  |  |  |  |
| Constant | 0.830 | 0.012 | 0.811 | 0.019 | 0.753 | 0.034 | 1.053 | 0.004 |
|  |  |  |  |  |  |  |  |  |
| **Abandoning** |  |  |  |  |  |  |  |  |
| Age class |  |  |  |  |  |  |  |  |
| 18-24 | -0.235 | 0.607 | -0.318 | 0.482 | -0.248 | 0.584 | -0.315 | 0.485 |
| 30-34 | -0.140 | 0.705 | -0.314 | 0.400 | -0.231 | 0.531 | -0.236 | 0.522 |
|  |  |  |  |  |  |  |  |  |
| Tertiary education | -0.287 | 0.344 | -0.186 | 0.542 | -0.233 | 0.445 | -0.238 | 0.431 |
|  |  |  |  |  |  |  |  |  |
| Married/cohabiting | -0.404 | 0.228 | -0.344 | 0.300 | -0.236 | 0.484 | -0.349 | 0.289 |
| With children | -0.539 | 0.147 | -0.512 | 0.162 | -0.648 | 0.081 | -0.524 | 0.151 |
| High spread COVID-19 region | 0.214 | 0.487 | 0.216 | 0.483 | 0.202 | 0.512 | 0.194 | 0.525 |
|  |  |  |  |  |  |  |  |  |
| Occupational status |  |  |  |  |  |  |  |  |
| NEET | 0.449 | 0.448 | 0.089 | 0.857 | 0.103 | 0.834 | 0.122 | 0.803 |
| Self-employed & temporary | 2.240 | 0.001 | 1.323 | 0.001 | 1.285 | 0.002 | 1.305 | 0.001 |
| October 2020 (ref. March 2020) | -0.629 | 0.092 |  |  |  |  |  |  |
| NEET*wave | -0.838 | 0.429 |  |  |  |  |  |  |
| self & temporary*wave | -2.133 | 0.023 |  |  |  |  |  |  |
|  |  |  |  |  |  |  |  |  |
| Financial condition already worsen |  |  | 0.781 | 0.044 |  |  |  |  |
| October 2020 (ref. March 2020) |  |  | -0.829 | 0.037 |  |  |  |  |
| Worse financial condition*wave |  |  | -0.610 | 0.365 |  |  |  |  |
|  |  |  |  |  |  |  |  |  |
| Income at risk |  |  |  |  | 0.660 | 0.082 |  |  |
| October 2020 (ref. March 2020) |  |  |  |  | -0.948 | 0.016 |  |  |
| Income at risk*wave |  |  |  |  | 0.032 | 0.964 |  |  |
|  |  |  |  |  |  |  |  |  |
| Occupation at risk |  |  |  |  |  |  | 0.089 | 0.809 |
| October 2020 (ref. March 2020) |  |  |  |  |  |  | -1.283 | 0.022 |
| Occupation at risk*wave |  |  |  |  |  |  | 0.281 | 0.677 |
|  |  |  |  |  |  |  |  |  |
| Constant | 0.176 | 0.647 | 0.059 | 0.883 | 0.039 | 0.925 | 0.305 | 0.471 |

*Text S1_1. The selection into the “no plans” group*

We might think that, even in January 2020 (before the occurrence of the pandemic), young people perceiving their income or occupation as uncertain – and so independently of the COVID-19 crisis – were less prone to plan the arrival of a child during the next 12 months. This would imply a selection in the sample of those answering about their revised fertility plans due to the COVID-19 emergency. In this case, in fact, they would represent a less vulnerable sub-sample in terms of perspective financial and occupational consequences of the economic crisis.

To explore whether the hypothesis of a sample selection based on perceived economic and occupational uncertainty is valid, we run a set of logistic models (see Table S1_6). In the Model 1 we include the occupational status, plus a set of control variables including only the socio-demographic characteristics and the residence in a region with a high diffusion of the COVID-19. In the Model 2, Model 3 and Model 4 we add, one by one, the other covariates related to the financial and the occupational conditions: respectively, the occupation perceived as at risk, the income perceived at risk and the actual impact of the crisis on the individual’s financial situation. We run separate models for women and men, to consider the possible different role of gender^[[1]](#footnote-1)^.

According to our selection hypothesis, the probability of being no-planners is higher when people perceive their income at risk, for both genders (Model 3) and for men also in the case in which they already experience an income loss (Model 4). However, when occupation is seen as vulnerable, this is associated with not having pre-covid fertility plans more on the female than on the male side (Model 2).

**Table S1_6. Logit models for the probability of not planning to have a child in the 2020, by including variables on income and occupation vulnerability due to COVID-19, in 2020 (Data source: Rapporto Giovani. March – October 2020).**

|  | **Model 1** | | | | **Model 2** | | | |
| --- | --- | --- | --- | --- | --- | --- | --- | --- |
|  | **Men** | | **Women** | | **Men** | | **Women** | |
|  | ***AME*** | ***p-value*** | ***AME*** | ***p-value*** | ***AME*** | ***p-value*** | ***AME*** | ***p-value*** |
| Age class |  |  |  |  |  |  |  |  |
| 25-29 | 0.099 | 0.003 | 0.031 | 0.364 | 0.100 | 0.003 | 0.031 | 0.354 |
| 30-34 | 0.030 | 0.356 | -0.005 | 0.891 | 0.029 | 0.370 | -0.005 | 0.891 |
|  |  |  |  |  |  |  |  |  |
| Tertiary education | -0.003 | 0.885 | 0.008 | 0.738 | -0.003 | 0.892 | 0.009 | 0.714 |
|  |  |  |  |  |  |  |  |  |
| Occupational status |  |  |  |  |  |  |  |  |
| NEET | 0.054 | 0.102 | 0.095 | 0.000 | 0.053 | 0.106 | 0.096 | 0.000 |
| self-employed & temporary | -0.087 | 0.019 | 0.036 | 0.298 | -0.088 | 0.017 | 0.036 | 0.305 |
|  |  |  |  |  |  |  |  |  |
| Married/cohabiting | -0.175 | 0.000 | -0.268 | 0.000 | -0.175 | 0.000 | -0.270 | 0.000 |
| With children | -0.053 | 0.106 | 0.026 | 0.345 | -0.053 | 0.111 | 0.027 | 0.336 |
| October 2020 (ref. March 2020) | 0.063 | 0.008 | 0.060 | 0.010 | 0.062 | 0.010 | 0.060 | 0.009 |
| High spread COVID-19 Region | 0.055 | 0.020 | 0.065 | 0.004 | 0.055 | 0.020 | 0.066 | 0.004 |
|  |  |  |  |  |  |  |  |  |
| Occupation at risk |  |  |  |  | 0.016 | 0.511 | 0.031 | 0.162 |
| Income at risk |  |  |  |  |  |  |  |  |
| Financial condition already worsen |  |  |  |  |  |  |  |  |
|  |  |  |  |  |  |  |  |  |
|  | **Model 3** | | | | **Model 4** | | | |
|  | **Men** | | **Women** | | **Men** | | **Women** | |
|  | ***AME*** | ***p-value*** | ***AME*** | ***p-value*** | ***AME*** | ***p-value*** | ***AME*** | ***p-value*** |
| Age class |  |  |  |  |  |  |  |  |
| 25-29 | 0.097 | 0.003 | 0.022 | 0.507 | 0.101 | 0.002 | 0.030 | 0.371 |
| 30-34 | 0.022 | 0.495 | -0.015 | 0.643 | 0.027 | 0.398 | -0.005 | 0.882 |
|  |  |  |  |  |  |  |  |  |
| Tertiary education | 0.004 | 0.875 | 0.011 | 0.644 | 0.007 | 0.784 | 0.008 | 0.728 |
|  |  |  |  |  |  |  |  |  |
| Occupational status |  |  |  |  |  |  |  |  |
| NEET | 0.044 | 0.191 | 0.090 | 0.000 | 0.050 | 0.130 | 0.095 | 0.000 |
| self-employed & temporary | -0.097 | 0.008 | 0.031 | 0.373 | -0.097 | 0.009 | 0.036 | 0.302 |
|  |  |  |  |  |  |  |  |  |
| Married/cohabiting | -0.161 | 0.000 | -0.265 | 0.000 | -0.169 | 0.000 | -0.268 | 0.000 |
| With children | -0.059 | 0.073 | 0.028 | 0.305 | -0.053 | 0.108 | 0.026 | 0.345 |
| October 2020 (ref. March 2020) | 0.079 | 0.001 | 0.074 | 0.002 | 0.073 | 0.002 | 0.061 | 0.010 |
| High spread COVID-19 Region | 0.053 | 0.025 | 0.063 | 0.005 | 0.058 | 0.014 | 0.065 | 0.004 |
|  |  |  |  |  |  |  |  |  |
| Occupation at risk |  |  |  |  |  |  |  |  |
| Income at risk | 0.127 | 0.000 | 0.079 | 0.000 |  |  |  |  |
| Financial condition already worsen |  |  |  |  | 0.095 | 0.000 | 0.005 | 0.826 |
|  |  |  |  |  |  |  |  |  |

**Robustness checks**

*Text S1_2. Stronger and weaker retrospective fertility intentions*

We test the differences in proportions (for categorical variables) and the differences in means (for continuous variables) across the enlarged and the restricted samples, contrasting them on our predictors and control variables.

Results from the tests show that there is no difference between the two groups in terms of age, gender, proportion of those having a tertiary degree, or living in a region with high or low spread of COVID-19 cases. However, those with stronger retrospective fertility intentions are more probably married (Pr_H0_=0.002) with at least one child (Pr_H0_=0.08), and with a “safer” occupational condition as they are more probably employees (Pr_H0_=0.005), while those with weaker intentions share a higher probability of being self-employed or temporary workers (Pr_H0_ = 0.02).

A further check has been done by performing the multinomial model on both the restricted and the enlarge samples. Results are reported in Table S1_7.

**Table S1_7 Multinomial models for the intention of still-planning, postponing or abandoning the pre-pandemic fertility plan, in the restricted and the enlarged samples (Data source: Rapporto Giovani COVID survey, March 2020 and October 2020)**

|  | **Restricted sample** | | **Enlarged sample** | |
| --- | --- | --- | --- | --- |
|  | **Coeff** | **p-value** | **Coeff** | **p-value** |
| **Still planning** |  |  |  |  |
| **Postponing** |  |  |  |  |
|  |  |  |  |  |
| Women | -0.128 | 0.556 | -0.187 | 0.300 |
|  |  |  |  |  |
| Age class |  |  |  |  |
| 18-24 | -0.039 | 0.909 | 0.074 | 0.788 |
| 30-34 | -0.227 | 0.386 | -0.343 | 0.106 |
|  |  |  |  |  |
| Tertiary education | -0.014 | 0.949 | 0.070 | 0.702 |
|  |  |  |  |  |
| Married/cohabiting | -0.696 | 0.004 | -0.608 | 0.002 |
| With children | -0.380 | 0.076 | -0.214 | 0.179 |
| October 2020 (ref. March 2020) | 0.454 | 0.055 | -0.452 | 0.011 |
| High spread SARS-CoV-2 region | -0.580 | 0.790 | -0.001 | 0.997 |
|  |  |  |  |  |
| Occupational status (ref. Employees) |  |  |  |  |
| NEET | -0.417 | 0.207 | 0.005 | 0.983 |
| self-employed & temporary | 0.232 | 0.482 | 0.354 | 0.192 |
|  |  |  |  |  |
| Constant | -0.329 | 0.489 | 1.338 | 0.000 |
|  |  |  |  |  |
| **Abandoning** |  |  |  |  |
|  |  |  |  |  |
| Women | 0.145 | 0.579 | 0.237 | 0.245 |
|  |  |  |  |  |
| Age class |  |  |  |  |
| 18-24 | -0.350 | 0.378 | -0.123 | 0.688 |
| 30-34 | -0.572 | 0.055 | -0.426 | 0.068 |
|  |  |  |  |  |
| Tertiary education | -0.045 | 0.868 | -0.422 | 0.049 |
|  |  |  |  |  |
| Occupational status (ref. Employees) |  |  |  |  |
| NEET | 0.318 | 0.350 | 0.379 | 0.149 |
| Self-emplyed& temporary | 0.600 | 0.114 | 0.877 | 0.002 |
|  |  |  |  |  |
| Married/cohabiting | -0.578 | 0.042 | -0.575 | 0.009 |
| With children | -0.185 | 0.440 | -0.173 | 0.322 |
| October 2020 (ref. March 2020) | -0.211 | 0.427 | -1.047 | 0.000 |
| High spread SARS-CoV-2 region | 0.015 | 0.955 | -0.168 | 0.441 |
|  |  |  |  |  |
| Constant | 0.022 | 0.967 | 1.625 | 0.000 |

*Text S1_3. Clustering occupational categories*

In order to provide further empirical evidence on the appropriateness of our categorization of the occupation vulnerability variable, we run the same model as reported in Figure 2 in the manuscript, by keeping all the occupational categories as much disaggregated as possible (see Figure S1_1). We pool men and women together, as well as the two waves, to have enough cases within each occupational category. The levels of the predicted probabilities, and especially those for the “abandoning” and “still-planning” outcomes, support our clustering decisions, by showing that fertility plans are quite homogeneous among temporary and permanent employees and professional/managers. On the same line, also self-employed and precarious workers are rather similar, and especially when abandoning the fertility plans.

**Figure S1_1. Predicted probabilities of changes or confirmations of pre-COVID fertility plans in March and October 2020 across occupational conditions (confidence intervals for pair-wise comparisons at the 10% significance level)**

Note: Predicted probabilities bases on a multinomial logit model, controlling for age class, education, marital status, presence of children. Full results are available upon request to the authors. Confidence intervals are calculated to allow for multiple comparisons of predicted probabilities, i.e., to allow testing their statistical equality at an approximate 5% significance level. Overlap between a pair of intervals indicate that the corresponding predicted probabilities are not statistically different at the 10% level, while non-overlap indicate a significant difference.

*Including full-time students in the analysed samples*

**Figure S1_2 Predicted probabilities of intending to have a(nother) child (answer: “surely yes”) in the next 12 months by occupational condition in 2016 and 2020 and by gender, including full-time students (confidence intervals for pair-wise comparisons at the 10% significance level)**

Note: Predicted probabilities bases on a generalized ordinal logit model, controlling for age class, education, marital status, presence of children, among those intending to have a child in the next 2 years (2020) or 3 years (2016). Full results are available in Table 1A in Supplementary Materials. Confidence intervals are calculated to allow for multiple comparisons of predicted probabilities, i.e., to allow testing their statistical equality at an approximate 5% significance level. Overlap between a pair of intervals indicate that the corresponding predicted probabilities are not statistically different at the 10% level, while non-overlap indicate a significant difference. When differences of interest are significant at the 5% level, this is noticed in the text.

**Figure S1_3 Predicted probabilities of changes or confirmations of pre-COVID fertility plans in March and October 2020 across occupational conditions, including full time students (confidence intervals for pair-wise comparisons at the 10% significance level)**

Note: Predicted probabilities bases on a generalized ordinal logit model, controlling for age class, education, marital status, presence of children, among those intending to have a child in the next 2 years (2020) or 3 years (2016). Full results are available in Table 1A in Supplementary Materials. Confidence intervals are calculated to allow for multiple comparisons of predicted probabilities, i.e., to allow testing their statistical equality at an approximate 5% significance level. Overlap between a pair of intervals indicate that the corresponding predicted probabilities are not statistically different at the 10% level, while non-overlap indicate a significant difference. When differences of interest are significant at the 5% level, this is noticed in the text.

**Figure S1_4 Predicted probabilities of changes or confirmations of pre-COVID fertility plans in March and October 2020 for those who experienced and those who did not experience a worsening in their financial situation, including full-time students (confidence intervals for pair-wise comparisons at the 5% significance level; March and October 2020 waves pooled)**

Note: Predicted probabilities bases on a multinomial logit model, controlling for age class, education, marital status, presence of children. Full results are available in Tables 2A and 3A in Supplementary Materials. Confidence intervals are calculated to allow for multiple comparisons of predicted probabilities, i.e., to allow testing their statistical equality at an approximate 5% significance level. Overlap between a pair of intervals indicate that the corresponding predicted probabilities are not statistically different at the 10% level, while non-overlap indicate a significant difference. No differences in terms of significancy has been found with confidence intervals at 10%.

**Figure S1_5 Predicted probabilities of changes or confirmations of pre-COVID fertility plans in March and October 2020 for those who perceived and those who do not perceived their future occupation as at risk, including full-time students (confidence intervals for pair-wise comparisons at the 5% significance level; March and October 2020 waves pooled)**

Note: Predicted probabilities bases on a multinomial logit model, controlling for age class, education, marital status, presence of children. Full results are available in Tables 2A and 3A in Supplementary Materials. Confidence intervals are calculated to allow for multiple comparisons of predicted probabilities, i.e., to allow testing their statistical equality at an approximate 5% significance level. Overlap between a pair of intervals indicate that the corresponding predicted probabilities are not statistically different at the 10% level, while non-overlap indicate a significant difference. No differences in terms of significancy has been found with confidence intervals at 10%.

**Figure S1_6 Predicted probabilities of changes or confirmations of pre-COVID fertility plans in March and October 2020 for those who perceived and those who do not perceived their future income as at risk, including full-time students (confidence intervals for pair-wise comparisons at the 5% significance level; March and October 2020 wave pooled)**

Note: Predicted probabilities bases on a multinomial logit model, controlling for age class, education, marital status, presence of children. Full results are available in Tables 2A and 3A in Supplementary Materials. Confidence intervals are calculated to allow for multiple comparisons of predicted probabilities, i.e., to allow testing their statistical equality at an approximate 5% significance level. Overlap between a pair of intervals indicate that the corresponding predicted probabilities are not statistically different at the 10% level, while non-overlap indicate a significant difference. No differences in terms of significancy has been found with confidence intervals at 10%.

1. We also performed the same analyses on the pooled sample of women and men, including interactions between the predictors and the gender dummy variable. However, results were often not enough statistically relevant to be interpreted. [↑](#footnote-ref-1)
